# Molecular nociceptive mechanisms in migraine: The migraine cascade

**DOI:** 10.1111/ene.16333

**Published:** 2024-06-18

**Authors:** Song Guo, Sarah Louise Christensen, Mohammad Al‐Mahdi Al‐Karagholi, Jes Olesen

**Affiliations:** ^1^ Danish Headache Center, Department of Neurology, Translational Research Center, Rigshospitalet‐Glostrup, Faculty of Health and Medical Sciences University of Copenhagen Glostrup Denmark; ^2^ Department of Neurology Zealand University Hospital Roskilde Denmark

**Keywords:** animal model, CGRP, experimental headache, human provocation model, migraine, migraine trigger, PACAP

## Abstract

**Objective:**

This review will explore the categorization of migraine‐provoking molecules, their cellular actions, site of action and potential drug targets based on the migraine cascade model.

**Methods:**

Personal experience and literature.

**Results:**

Migraine impacts over 1 billion people worldwide but is underfunded in research. Recent progress, particularly through the human and animal provocation model, has deepened our understanding of its mechanisms. This model have identified endogenous neuropeptides such as calcitonin gene‐related peptide (CGRP) and pituitary adenylate cyclase‐activating peptide (PACAP) that induces controlled migraine‐like attacks leading to significant discoveries of their role in migraine. This knowledge led to the development of CGRP‐inhibiting drugs; a groundbreaking migraine treatment now accessible globally. Also a PACAP‐inhibiting drug was effective in a recent phase II trial. Notably, rodent studies have shed light on pain pathways and the mechanisms of various migraine‐inducing substances identifying novel drug targets. This is primarily done by using selective inhibitors that target specific signaling pathways of the known migraine triggers leading to the hypothesized cellular cascade model of migraine.

**Conclusion:**

The model of migraine presents numerous opportunities for innovative drug development. The future of new migraine treatments is limited only by the investment from pharmaceutical companies.

## INTRODUCTION

Migraine is the second most disabling of all diseases affecting more than 1 billion people worldwide [1]. The National Institutes of Health (NIH) in the USA fund very few research projects in migraine, and a report on NIH funding showed that migraine is one of the least‐funded research areas relative to its very high burden [2]. Also, a large European study demonstrated that, in relation to socioeconomic costs, headache disorders were the least funded [3]. In Europe the cost of migraine has been estimated to range between 50 and 100 billion Euros per year [4], and similar figures have been suggested in the United States [5].

Despite limitations in funding, the progress in our understanding of migraine mechanisms has been impressive. Much of the increased understanding derives from the use of a human provocation model of migraine [6]. It involves double blind administration of a presumed headache‐inducing substance or placebo and repeated scoring of headache and other migraine characteristics in a controlled setup. The human provocation model is unique for migraine and not used in other diseases. It could be used in migraine research for two reasons. First, migraine occurs in attacks and individuals with migraine usually report no migraine‐associated symptoms between attacks. Therefore, attacks can be provoked. Secondly, although attacks are very painful, they are transient, treatable and cause no damage. Provocation is therefore ethically fully acceptable. The model first used glyceryl trinitrate (GTN), a pro‐drug for nitric oxide (NO) [7]. GTN caused headaches in healthy volunteers and migraine attacks in individuals with migraine. Next, and importantly, calcitonin gene‐related peptide (CGRP) also caused headache in healthy volunteers and migraine attacks in individuals with migraine [8]. Together with other evidence, this was crucial for the pharmaceutical development of small molecule CGRP receptor blocking drugs and antibodies against CGRP or its receptor. These drugs are now on the market worldwide and promise to revolutionize the treatment of migraine. Pituitary adenylate cyclase‐activating peptide (PACAP) is another provoking substance where an antibody was recently effective in migraine [9]. But the human model has delivered many other migraine‐provoking molecules such as vasoactive intestinal polypeptide (VIP), phosphodiesterase‐5 (PDE5) inhibitor sildenafil, PDE3 inhibitor cilostazol, histamine, prostanoids and potassium channel openers [6].

The human studies have inspired several rodent models of migraine that are validated against the highly specific migraine drugs sumatriptan and CGRP antagonists. They can elucidate several important questions, for example whether antagonists are effective, whether migraine pain originates within or outside of the brain, where the nociception of migraine occurs, and which cell type(s) is the most important. Preclinical models also analyze how signaling cascades interact. The aims of the present personal view are to categorize the molecules that cause migraine, discuss their possible anatomical site of action, the cell types involved, and the biochemical cascade caused by these provoking agents. Finally, possible drug targets derived from the migraine cascade model are suggested.

## METHOD OF LITERATURE SEARCH

PubMed was searched for articles published in English with use of the search terms “migraine”, “experimental headache”, “migraine or headache animal model”, “migraine trigger”, “human headache model” and “migraine provocation” amongst others. No publication date restrictions were applied. Papers from the authors' own files and from references cited in relevant articles were also identified. Reviews were chosen when space did not permit a more comprehensive treatment of a topic. The final reference list was generated based on articles' relevance to the topic of this personal view.

## MOLECULES THAT PROVOKE MIGRAINE

**FIGURE 1:**
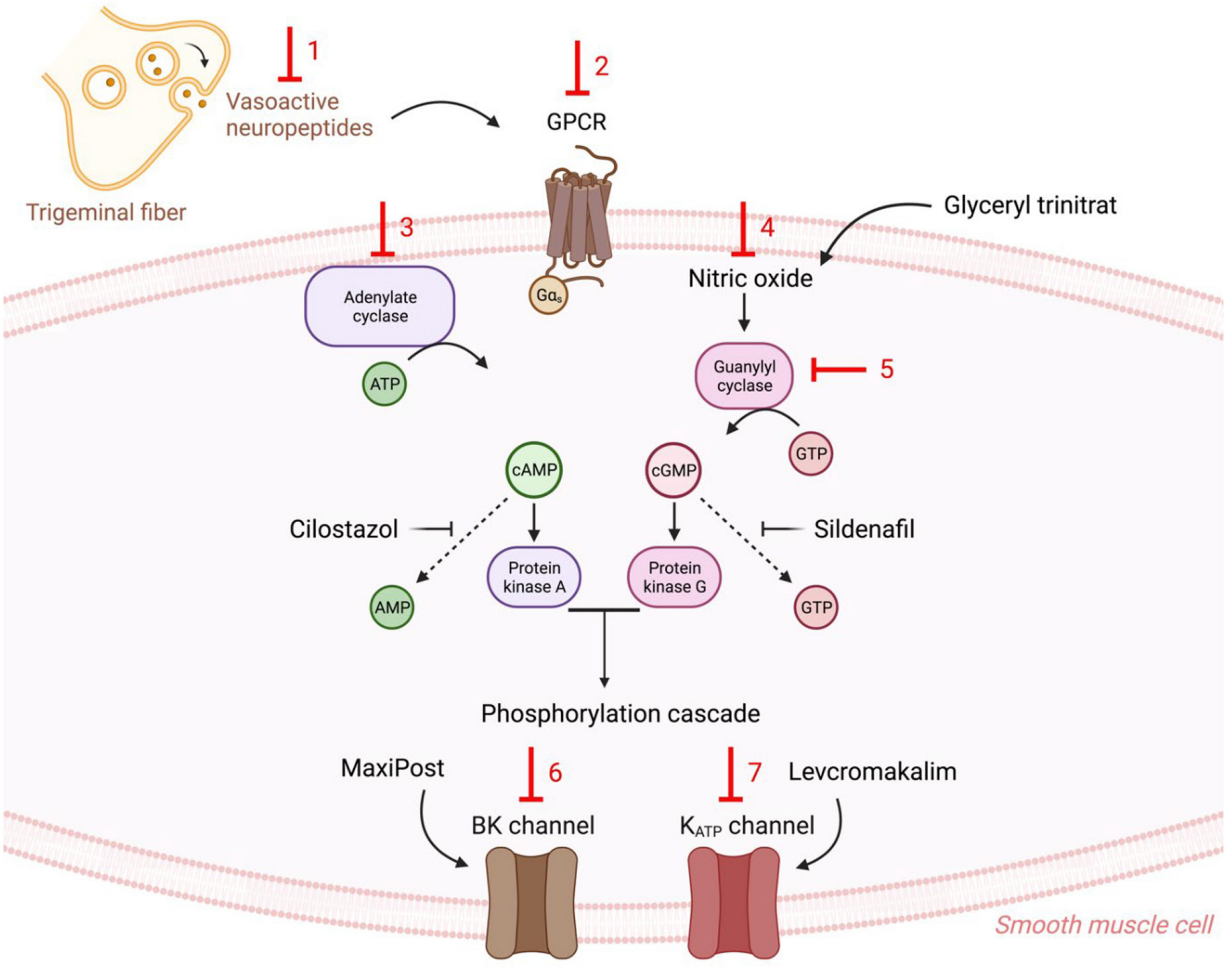
Levels of action and signaling cascades. Migraine‐provoking substances fall into three groups according to mode of action. One group includes vasoactive neuropeptides (adrenomedullin, amylin, CGRP, histamine, PACAP, prostanoids and VIP), which act on cell surface GPCR leading to upregulation of the second messengers cAMP and cGMP. The second group includes cilostazol, glyceryl trinitrate and sildenafil which act intracellularly by upregulation of the second messengers. The third group includes MaxiPost and levcromakalim, which open downstream potassium channels. Potential drug targets are marked with red. BK channel, large (big) conductance calcium‐activated potassium channels; cAMP, cyclic adenosine monophosphate; cGMP, cyclic guanosine monophosphate; CGRP, calcitonin gene‐related peptide; GPCR, G‐protein coupled receptors; K_ATP_ channel, ATP‐sensitive potassium channel; PACAP38, pituitary adenylate cyclase activating polypeptide 38; VIP, vasoactive intestinal peptide.

Migraine‐provoking substances fall into three groups according to mode of action (Figure 1). One group act on cell surface G‐protein coupled receptors (GPCR). It includes CGRP, PACAP, histamine, prostanoids and, more recently, amylin, VIP and adrenomedullin [6, 10, 11, 12, 13]. Stimulation of these receptors upregulates the second messenger cyclic adenosine monophosphate (cAMP) which activates protein kinase A and has many different actions one of which is phosphorylation of potassium channels which activates them. Activation of potassium channels may lead to hyperpolarization of smooth muscle cells [14] that prevents the opening of depolarization‐activated Ca^2+^ channels. This causes a decrease in Ca^2+^ entry to the cell leading to vasodilatation [15] and eventually neuroinflammation causing migraine pain [16].

The second group bypasses the cell membrane and acts directly intracellularly by accumulating the second messenger cAMP or cyclic guanosine monophosphate (cGMP). Cilostazol is a typical group 2 provoking agent [17]. It inhibits PDE3 and thus causes accumulation of cAMP. Sildenafil, also a typical group 2 provoking agent, inhibits PDE5 leading to accumulation of cGMP. The third group of provocative agents opens potassium channels. So far studied, are the selective opener of the ATP sensitive potassium (K_ATP_) channel, levcromakalim [18], and MaxiPost which is a selective opener of large (big) conductance calcium‐activated potassium (BK_Ca_) channels [19].

**FIGURE 2:**
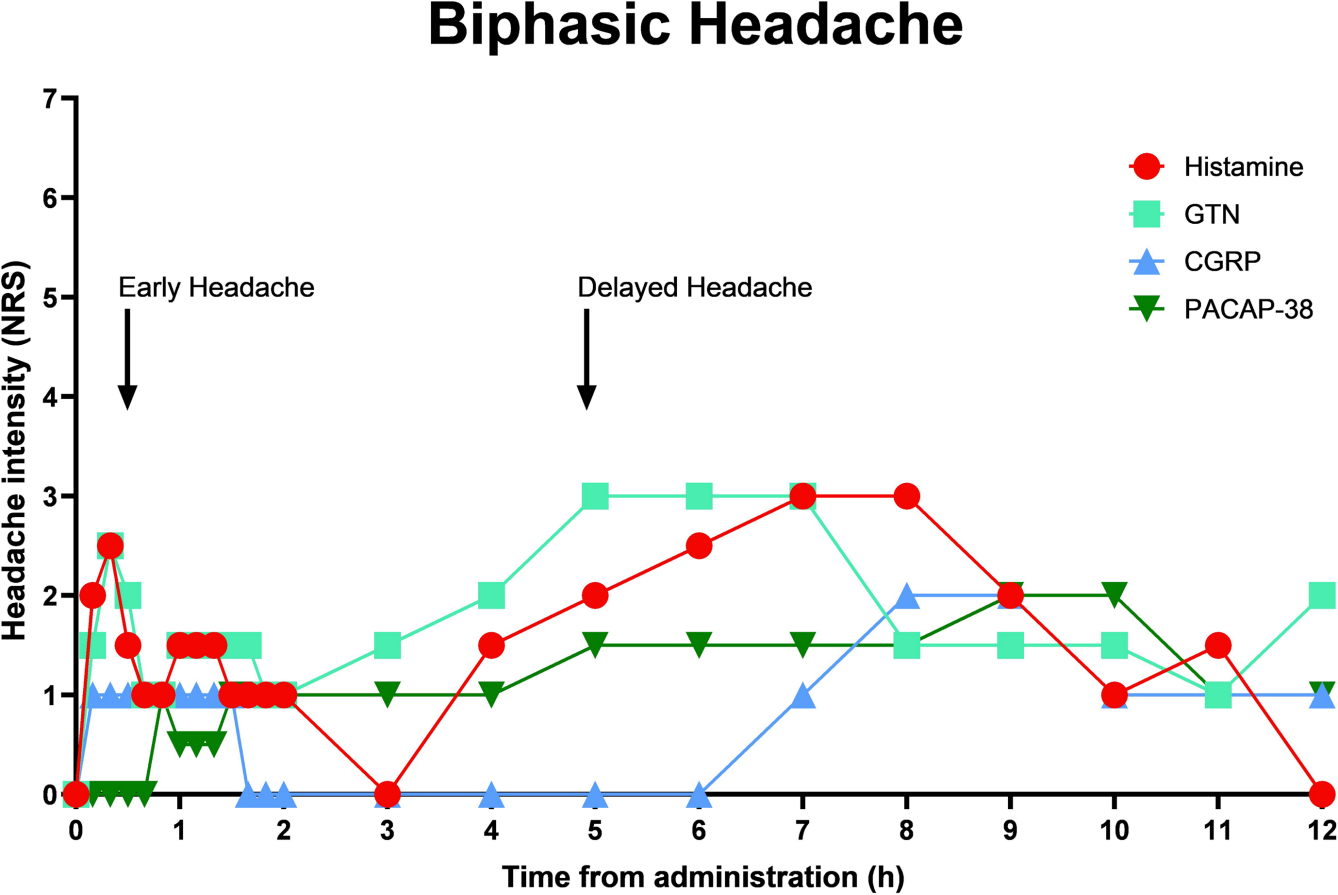
Provoked biphasic headache upon administration to migraine patients of headache‐inducing compounds in migraine patients but not in healthy controls. GTN and vasoactive peptides provoke in migraine patients an immediate headache followed by a delayed headache that fulfills the diagnostic criteria for a provoked migraine attack. CGRP, calcitonin gene‐related peptide; GTN, glyceryl trinitrate; PACAP38, pituitary adenylate cyclase activating polypeptide 38. The data of this figure have been taken from references 8, 39, 41, 71.

In healthy volunteers the headache response to all provoking substances is immediate, short lasting and monophasic. In individuals with migraine, GTN, histamine, CGRP and PACAP cause an immediate headache followed by a delayed headache a number of hours later that fulfills the diagnostic criteria for a migraine attack [6] (Figure 2). These membrane receptor ligands may need time to activate the intracellular processes leading to delayed migraine, hence the biphasic headache response. GTN easily crosses cell membranes and produces NO intracellularly. It sets in motion a cascade of biochemical reactions including activation of nitric oxide synthase, a well‐documented but unusual feedforward mechanism [20]. This may explain the biphasic headache induction shown in Figure 2. For all substances sharing a biphasic headache response the important question is what happens in the lag phase between the immediate headache and the delayed migraine. Unfortunately, it is difficult to study because it is seen only in those with migraine.

**FIGURE 3:**
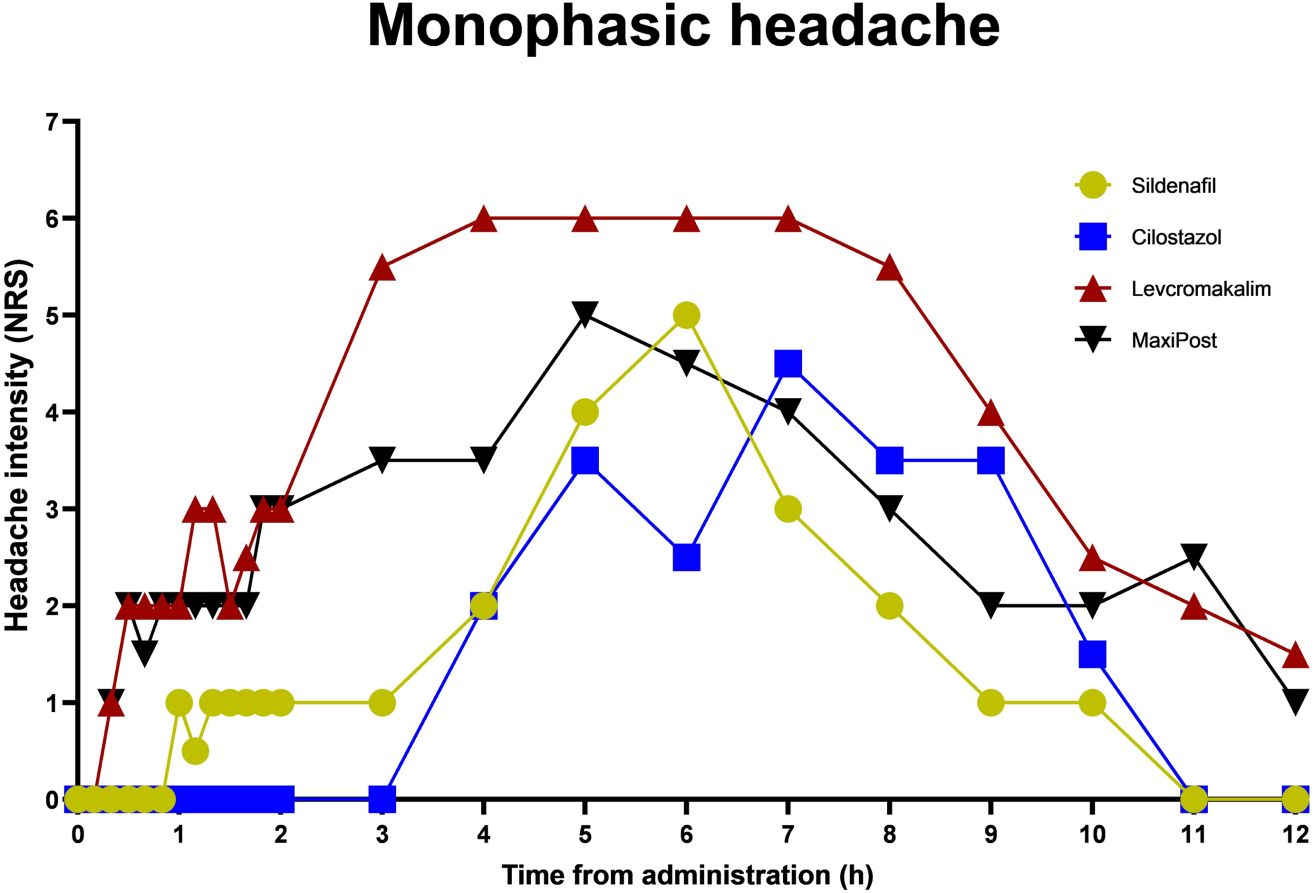
Provoked monophasic headache upon administration to migraine patients of headache‐inducing compounds. Cilostazol, sildenafil, levcromakalim and MaxiPost provoke in migraine patients a slowly rising monophasic headache response resulting in a migraine attack. The data of this figure have been taken from references 17, 18, 19, 42.

Other provoking substances cause a monophasic response not only in healthy volunteers but also in individuals with migraine. These substances are cilostazol, sildenafil and K_ATP_ and BK_Ca_ opening substances, levcromakalim and MaxiPost (Figure 3) [6, 18, 19]. Part of the explanation of the monophasic response is, perhaps, that cilostazol and sildenafil are administered orally and therefore have a protracted absorption, but increased second messenger concentration may also induce headache faster than activation of membrane receptors. Levcromakalim and MaxiPost are given as an infusion and, nevertheless, cause a monophasic headache response fulfilling the diagnostic criteria for an experimental migraine attack after a median of 3 h. Generally, group 2 provoking agents seem to be more potent (induction rate 83%–86%) than group 1 provoking agents (induction rate 57%–80%) [6], whereas group 3 agents are seemingly the most potent migraine triggers (induction rate 95%–100%) [18] (Table 1).

**TABLE 1:** Overview of some migraine‐provoking compounds that are tested or untested in behavioral in vivo mouse models of migraine using Von Frey testing for cutaneous hypersensitivity or light–dark box for photophobia.

	GTN	CGRP	PACAP38	Amylin	Cilostazol	Sildenafil	Levcromakalim
Sensitizes mice	Yes [24, 25, 26]	Yes [25, 27, 28]	Yes [29, 30]	Yes [12]	Yes [31]	[26]	Yes [32]
Triptan response	Yes [25, 33]	Untested	No [29]	Untested	Untested	Untested	Untested
Olcegepant response	Yes [25, 34]	Yes [35]	Untested	Untested	(Yes) [36]	Untested	(Yes) [36]
CGRP antibody response	Yes [28]	Yes [28, 37]	No [30]	Untested	Yes [36]	Untested	Yes [36]
PACAP antibody response	No [29]	No [30]	Yes [29, 30]	Untested	Untested	Untested	No [29]
Glibenclamide response	Yes [32]	Untested	Yes [38]	Untested	Yes [36]	Untested	Yes [32]
L‐NAME response	Yes (UP)	Untested	Untested	Untested	Untested	Untested	Yes (UP)
Migraine induction rate in humans (%)	80% [39]	57% [40]	58% [41]	41% [12]	86% [17]	83% [42]	100% [18]
Effect in humans of the inhibitor	Yes [22]	Yes [23]	Yes [9]	Untested	Untested	Untested	Untested

*Note*: For simplicity, models using rats are not included. Many more migraine‐provoking compounds await to be tested such as VIP, adrenomedullin, prostaglandin E_2_, MaxiPost and histamine.

Abbreviation: UP, unpublished.

## ACTION AND INTERACTION OF MIGRAINE‐PROVOKING MOLECULES IN BEHAVIORAL MOUSE MODELS

Rodent models play an indispensable role in the field of drug development due to their contributions in predicting drug efficacy, unraveling underlying mechanisms, identifying new drug targets and understanding their site of action. The use of in vivo mouse models to evaluate behaviors relevant to migraine is growing, offering significant insights into pain pathways [21]. In our opinion, the most validated of these behavioral models include the Von Frey method for assessing cutaneous tactile hypersensitivity and the light–dark box method for assessing photophobia [21]. How these models translate to the human experience of pain is difficult to judge, but four effective migraine drugs that inhibit the effect of migraine‐provoking molecules have been identified: non‐steroidal anti‐inflammatory drugs (NSAIDs) which inhibit prostanoids, N(G)‐monomethyl l‐arginine which inhibits NO production [22], CGRP‐antagonizing drugs and PACAP‐inhibiting antibodies [9, 23]. These anti‐migraine drugs and sumatriptan have shown efficacy in at least one behavioral mouse model. This demonstrates that the mouse models have some predictive value for therapeutic efficacy [9, 23]. K_ATP_ opener levcromakalim effectively provokes migraine but human antagonists of the relevant subtype channel Kir6.1SUR2B are not available. However, the efficacy of K_ATP_ channel antagonists was demonstrated in rat and mouse paving the road for human drug development [21]. There are many more migraine‐provoking compounds that await thorough testing in behavioral rodent models and perhaps in humans (Table 1).

Rodent models are particularly valuable for exploring the mechanisms of signaling pathways associated with migraine. Migraine‐inducing molecules like CGRP and PACAP, found in the trigeminal ganglion, interact by activation of sensory neurons that trigger the trigeminovascular system [43]. Recent studies using mouse models revealed an independent and distinct PACAP38 pathway, not reliant on CGRP‐, GTN‐ or levcromakalim‐induced migraine pathways [29, 30]. PACAP38 antagonism may therefore be a novel treatment for patients unresponsive to CGRP inhibitors or other migraine‐specific drugs.

Finally, animal models are also crucial in determining the site of action of new drugs which is difficult in humans. CGRP monoclonal antibodies for the treatment of migraine have been marketed without consensus about their site of action.

## SITE OF MIGRAINE PROVOCATORY ACTION

The anatomical site of migraine provocatory action should be examined in context with the fact that all migraine‐provoking drugs cause cephalic pain only and no pain in any other part of the body. Migraine may be a brain disorder in the sense that an attack may often start in the brain [44], but it leads to peripherally ignited nociception. The strongest arguments for a peripheral origin of migraine pain (nociception) are the fact that migraine attacks can be induced and also alleviated by substances that do not cross the blood–brain barrier [6]. All substances that can trigger a migraine attack in humans are extracerebral vasodilators and most (CGRP, PACAP, adrenomedullin, amylin) do not cross the blood–brain barrier [45]. Regarding therapy, triptans cross the blood–brain barrier poorly and this is even more so for the novel small molecule CGRP receptor antagonists. Antibodies against CGRP or its receptor are primarily used as prophylactics but hardly cross the blood–brain barrier. Eptinezumab, an antibody against CGRP, is even effective within the first hours of administration in humans and in rodent models [46]. CGRP antibodies therefore act peripherally possibly by modulating the interaction between C‐type and Aδ‐type sensory neurons in the trigeminal ganglion or trigeminal nerve fibers [23]. They may also block CGRP responses in the dura mater. Likewise, the most effective acute migraine drugs, the triptans, constrict extracerebral arteries simultaneously with relief of migraine pain, which also suggests a peripheral site of action [47]. Preclinical models indicate the site of action for triptans to be in the first‐order neuron localized outside of the blood–brain barrier [48]. Intracerebroventricular administration of CGRP antagonists had no effect in a validated mouse model whilst systemic administration alleviated hypersensitivity induced by GTN [49]. Accordingly, the central neuronal PACAP receptor, PAC_1_, mediated delayed activation and sensitization of trigeminocervical neurons in rats [50], but anti‐PAC_1_‐receptor antibodies failed to show therapeutic efficacy in humans [51]. This indicates that some of the migraine‐inducing substances have different effects inside and outside of the brain.

**FIGURE 4:**
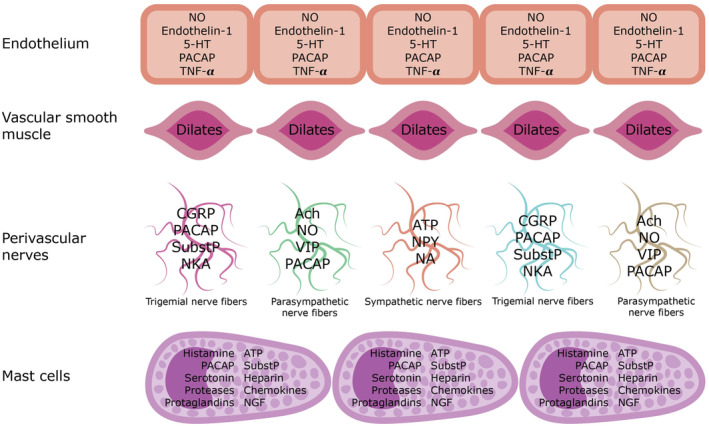
Cells possibly involved in migraine nociception. Endothelial and mast cells can liberate nociceptive molecules and terminal nerve fibers likewise causing cross‐talk between nerve types. Smooth muscle relaxation is associated with increased extracellular potassium concentration, also nociceptive. The finer details of how this complete system interacts remain to be clarified. 5‐HT, 5‐hydroxytryptamine receptors; Ach, acetylcholine; ATP, adenosine triphosphate; CGRP, calcitonin gene‐related peptide; NA, noradrenaline; NGF, nerve growth factor; NKA, neurokinin A; NO, nitric oxide; NPY, neuropeptide Y; PACAP38, pituitary adenylate cyclase activating polypeptide 38; SubstP, substance P; TNF‐α, tumor necrosis factor α; VIP, vasoactive intestinal peptide.

The perivascular nerves form a dense network full of signaling molecules which alone or, more likely in combination, can sensitize/or activate sensory afferents (Figure 4). Stimulation or distension of the major cerebral arteries has resulted in pain that was associated with nausea or vomiting [52], which are common accompanying symptoms of migraine. Moreover, physical activity and the accompanying increase in arterial pulsations almost always aggravate migraine pain and frequently transform it from constant to pulsating. Lastly, the pulsing pattern of migraine in 80% of patients and the localization of experimentally produced pain to extracranial and intracranial arteries are further arguments for arterial pain in migraine [52]. Thus, it is concluded that periarterial nerves are likely to be the primary source of migraine nociception. The mechanism for signaling from vasodilatation to sensory nerves leading to pain perception remains to be fully elucidated. In vascular smooth muscle cells, the activation of K_ATP_ and BK_Ca_ channels causes a substantial potassium efflux which perhaps is directly nociceptive [53]. Recently, it has been suggested that mechanosensitive Piezo channels and TRPM3, found in both neurons and vessels in the meninges and the trigeminovascular system, may contribute to the further signaling of migraine pain [54]. These channels may be activated by vasodilatation, increased pulse waves and shear stress. This activation potentially triggers nociceptive neuronal firing and prompts mast cell degranulation [54], which may result in a heightened release of pro‐inflammatory and pro‐nociceptive compounds.

## WHICH CELL TYPES ARE INVOLVED IN THE BIOCHEMICAL CASCADE OF MIGRAINE?

The cell type most obviously involved is the vascular smooth muscle because both spontaneous and provoked attacks are associated with vasodilatation (Figure 4). Evidence discussed above and evidence from a targeted knockout mouse model of migraine suggest that it is blood vessels outside the brain [55]. Endothelial cells also harbor the described mechanisms due to their ability to release various pro‐inflammatory mediators such as NO that is synthesized by endothelial cells [7]. Neurons are different because activation of neurons with the above‐mentioned signaling molecules hyperpolarizes the cell. This is particularly evident with K_ATP_ channel openers. They have even been evaluated for their possible analgesic efficacy. In a mouse model, opening of K_ATP_ channels in the brain achieved by intraventricular infusion of levcromakalim resulted in an increased pain threshold on the hot plate [56]. Conversely, it caused hypersensitivity when given systemically. It seems highly unlikely that the K_ATP_ channel opening in neurons plays a role in migraine nociception.

The role of glial cells in migraine is a growing research area, particularly regarding their involvement in neuroinflammation and neuronal regulation in the central nervous system, but also in the peripheral nervous system via satellite glia and Schwann cells. During migraine attacks, activated glial cells may cause glial–neuron cross‐talk by producing pro‐inflammatory cytokines, sensitizing trigeminal neurons and intensifying pain [57]. Abnormal glial cell activity could disrupt neurotransmitter regulation and ion homeostasis, contributing to cortical spreading depression associated with migraine with aura but not migraine without aura [58]. Also, glial cells maintain endothelial integrity of the blood–brain barrier.

Mast cells may be implicated in migraine via several potential pro‐inflammatory mechanisms due to their meningeal localization and proximity to trigeminal nerve fibers. Activated mast cells release mediators like histamine, tryptase and cytokines, inducing vasodilatation, increased vascular permeability, endothelial dysfunction and pain sensitization [59]. Mast cell degranulation contributes to PACAP‐induced vasodilatation [60]. The mast cells may further exacerbate neurogenic inflammation by releasing pro‐inflammatory mediators in response to neuropeptides such as CGRP and PACAP [61].

## A CELLULAR MODEL OF MIGRAINE

Even without knowing the exact cell type(s) involved, it is possible to model the involved biochemical cascade. Activation of membrane receptors stimulates adenyl cyclase via G proteins to produce second messenger cAMP. NO stimulates formation of cGMP. These second messengers, via their respective protein kinases, phosphorylate and open potassium channels. On this basis a cellular model has been proposed [62]. Our expanded version of this model is shown in Figure 1.

Regarding the cAMP pathway it was recently shown that cilostazol‐induced migraine cannot be blocked by a CGRP receptor antibody [63]. This suggests the role of cAMP as a common pathway on which several mechanisms can act. But it does not prove that CGRP works via cAMP in migraine. It should be shown that blocking cAMP production abolishes the pain‐inducing effect of CGRP and such an experiment has not been done either in humans or in animals. Inhibition of the effect of each provoking substance in future will improve our understanding of migraine mechanisms and of possible new drug targets. Similar blocking experiments in man or animal are needed regarding the next step, protein kinases. Thus, there is a logic in the model shown in Figure 1 and there is clinical scientific support of the pathway [62]. Several K_ATP_ channel agonists have been developed for clinical practice including levcromakalim, nicorandil, tifenazoxide, pinacidil, minoxidil and diazoxide. It has been commonly reported that all these K_ATP_ channel agonists induce headache [64]. It is particularly noteworthy that the most prevalent side effect associated with the use of levcromakalim, nicorandil and pinacidil was headache.

Blocking of ion channels should result in inhibition of migraine provocation. That would prove the final step of the cascade. Here, animal experiments come to the rescue because glibenclamide, which blocks K_ATP_ channels non‐selectively, is effective in preventing migraine‐associated responses by cilostazol and nitroglycerin in rat and mouse models [36, 65]. The same is true in mice where the Kir6.1 subunit of the K_ATP_ channel has been knocked out in smooth muscle cells [56]. Other provoking substances should also be tried using this paradigm. Overall, these findings indicate the involvement of K_ATP_ channels in migraine pathophysiology, identifying them as one of the most recent and promising molecular targets for migraine treatment.

No migraine‐provoking substance activates a membrane receptor coupled to soluble guanylyl cyclase. It is likely, however, that NO causes migraine via cGMP and not via its many other actions. Human studies in both healthy volunteers and individuals with migraine demonstrate that increasing cGMP by the PDE5 inhibitor sildenafil induces headache respectively migraine with the same efficacy as NO donors [42]. Direct pharmacological activation of soluble guanylyl cyclase results in migraine relevant hypersensitivity in a mouse model whilst inhibition of soluble guanylyl cyclase prevents hypersensitivity induced by NO donors [66]. There are no studies of cGMP antagonists but inhibition of cGMP degradation by sildenafil effectively caused hypersensitivity in mice [67]. There are no experiments on protein kinase G inhibition but blocking K_ATP_ channels with glibenclamide abolished hypersensitivity induced by an NO donor and cAMP elevation in a mouse model of migraine [36]. Natriuretic peptides which include atrial natriuretic peptide, brain natriuretic peptide and C‐type natriuretic peptide activate the cGMP‐dependent pathway via particulate guanylyl cyclase in cerebral arteries, but they were ineffective as headache provokers in healthy volunteers [68]. It is possible that the headache‐inducing effect of natriuretic peptides is dose‐dependent, but very high doses are not possible due to severe side effects.

On balance, it seems likely that the model shown in Figure 1 is real. But there are missing links in the final proof of this cascade of events. Probably, the cAMP system is either dominant or perhaps the only one of real significance. This is possible because there is evidence of cross‐over mechanisms from cGMP to cAMP [69].

## POSSIBLE DRUG TARGETS IN THE CASCADE MODEL

Human and animal research that has led to an understanding of the intracellular biochemical cascade of migraine opens unforeseen opportunities for migraine drug development. It is immediately clear that, like CGRP antagonistic treatments already benefiting millions of migraine sufferers, antagonism of PACAP is also effective. NSAIDs block the production of all prostanoids and are effective in migraine [70]. Perhaps selective blockers of prostanoid receptors will prove better or have fewer side effects. Inhibitors of other migraine‐provoking agents should also be tested in migraine models and then in patients. Downstream from the membrane receptors there are further possibilities as outlined by the arrows in Figure 1. Adenylyl cyclase inhibition is one. There are also chemical antagonists of cAMP and inhibitors of protein kinase A. Activation of phosphodiesterase subtypes to speed up the degradation of cAMP and/or cGMP is possible. Finally, blocking K_ATP_ and BK_Ca_ channels seems to be promising future drug targets. In the NO triggered cascade, the first step to be inhibited is the formation of NO from l‐arginine. Here, the primary co‐factor tetrahydrobiopterin acid can be inhibited. Furthermore, the NOS enzymes eNOS and nNOS are obvious drug targets. Soluble guanylyl cyclase can be inhibited pharmacologically, or its breakdown can be accelerated. Protein kinase G can be inhibited and, finally, the K_ATP_ channel and the BK_Ca_ channel can be blocked. Thus, the cellular model of migraine is full of possibilities for novel development of drugs for migraine treatment and with modern technology it may be possible to target some of these at relevant sites specifically to avoid side effects. Only the necessary investment by pharma companies limits the future options for new migraine therapeutics.

## CONCLUSION

Several naturally occurring signaling molecules/mechanisms can induce attacks in individuals with migraine. The action takes place outside of the blood–brain barrier, most likely in vascular smooth muscle or endothelium leading to activation of perivascular trigeminal nerve fibers. Antagonists to these provoking mechanisms are likely drug candidates like the marketed CGRP antagonists. But a plethora of other mechanisms are validated and only await initiative from the pharma industry.

## AUTHOR CONTRIBUTIONS

Song Guo: writing—review and editing; writing—original draft; conceptualization; methodology; data curation. Sarah Louise Christensen: writing—review and editing; writing—original draft; conceptualization. Mohammad Al‐Mahdi Al‐Karagholi: writing—original draft; writing—review and editing; visualization. Jes Olesen: conceptualization; supervision; writing—review and editing; writing—original draft; data curation; methodology.

## CONFLICT OF INTEREST STATEMENT

Jes Olesen owns the start‐up company Cephagenics. All other authors report no competing interests.

## Data Availability

Data sharing does not apply to this article since no new data were generated or examined in this study.
